# Fibroblast-Like Cells Differentiated from Adipose-Derived Mesenchymal Stem Cells for Vocal Fold Wound Healing

**DOI:** 10.1371/journal.pone.0092676

**Published:** 2014-03-24

**Authors:** Rong Hu, Wei Ling, Wen Xu, Demin Han

**Affiliations:** 1 Department of Otorhinolaryngology-Head Neck Surgery, Beijing Tongren Hospital, Capital Medical University, Beijing, The People's Republic of China; 2 Department of Anatomy, Capital Medical University, Beijing, The People's Republic of China; The University of Hong Kong, Hong Kong

## Abstract

Tissue engineering has revealed the potential to regenerate injured vocal folds, and identification of the most suitable seed cells has remained a hot topic of research. The aim of this study was to implant fibroblast-like cells differentiated from adipose-derived mesenchymal stem cells (ADSCs) in a canine acute vocal fold wound model. We then sought to characterize changes in the extracellular matrix (ECM) proteins of vocal fold lamina propria. For this purpose, ADSCs were induced to differentiate into fibroblasts under the regulation of connective tissue growth factor in vitro. Cell surface proteins were identified by immunofluorescence staining. Thirty vocal folds of 17 canines were injured by localized resection and injected with fibroblast-like cells (differentiated ADSCs, dADSCs), ADSCs or vocal fold fibroblasts (VFFs). The morphology of vocal folds was observed, and the characteristics of ECM protein components (collagen, elastin, hyaluronic acid, decorin and fibronectin) were evaluated by immunofluorescence staining from 15 days to 6 months following implantation. The results showed that in vitro, the dADSCs showed morphology and cell surface protein expression similar to those of VFFs. After implantation in vivo, the surfaces of the recipient vocal folds became almost smooth in the dADSCs and ADSCs groups at 6 months but remained slightly concave and stiff in the VFFs group. The elastin fluorescence intensity increased significantly and was maintained at a high level in the dADSCs group. The collagen fluorescence intensity increased slightly in the dADSCs and ADSCs groups, whereas it demonstrated a more irregular arrangement in the VFFs group. The fluorescence intensity of hyaluronic acid, decorin and fibronectin was similar between the three implanted groups. These results indicated that dADSCs may confer an advantage for vocal fold wound healing. Furthermore, dADSCs have the ability to secrete ECM components in vivo, particularly elastin, which may be beneficial for vocal fold vibration recovery.

## Introduction

The delicate composition and orderly arrangement of the vocal fold lamina propria extracellular matrix (ECM) is the foundation of its delicate vibration. Vocal fold injury leads to changes in the ECM composition and distribution in the lamina propria and the development of significant fibrous hyperplasia, disorderly deposition and local contracture, which is an irreversible process that complicates treatment [1]–[3]. At present, with the development of tissue engineering techniques, injuries to the vocal folds have revealed the potential to regenerate in the appropriate environment both in vitro and in vivo [4]–[20].

Cells, scaffolds and regulatory factors are the three key elements in tissue engineering focusing on complex construction. The selection of seed cells is a hot spot for research. In a previous study [17], we demonstrated a satisfactory effect of adipose-derived mesenchymal stem cells (ADSCs) on vocal fold regeneration. However, the in vivo growth and differentiation characteristics of stem cells remain unclear, and certain tumorigenic risks also exist.

Fibroblasts have been widely used in tissue engineering studies on blood vessels and skin due to their ability to secrete ECM proteins and aid in wound healing [21], [22]. Some researchers have used cultured fibroblasts from the buccal mucosa, skin or vocal folds to promote the healing of injured vocal folds [12]–[16]. However, the ability of cultured fibroblasts to secrete ECM proteins in vivo is not guaranteed after implantation. In addition, the characteristics of fibroblasts from the buccal mucosa and skin are different from those of vocal fold fibroblasts (VFFs), and the extraction of autologous VFFs damages the normal vocal fold lamina propria tissue.

Therefore, we expect that the development of a simple and stable source of differentiated cells as seed cells would be useful for vocal fold wound healing. Some researchers have reported that mesenchymal stem cells can be induced into fibroblast-like cells under the appropriate in vitro conditions and also have the ability to secrete ECM proteins. Recently, these cells have also been used as seed cells for the repair of ligaments and the skull with favorable efficiency [23], [24]. However, they have not been used to treat injured vocal folds in vivo. Thus, the capacity of fibroblast-like cells differentiate from ADSCs (differentiated ADSCs, dADSCs) for vocal fold wound healing requires further study.

In this study, we implanted autologous dADSCs in hyaluronic acid gel scaffolds in a canine acute vocal fold injury model. We focused on characterizing the features of the dADSCs in vitro and the features of vocal fold wound healing after dADSCs implantation in vivo. We also characterized and compared the vocal fold ECM proteins after the implantation of three types of cells: dADSCs, ADSCs and VFFs.

## Materials and Methods

The study included 17 healthy experimental canines (license permit: SCXK 2009–0014) (1.5-2 years of age) weighing 17.3–20.8 kg. The animals were treated in strict accordance with the National Institute of Health Guide for the Care and Use of Laboratory Animals, and their experimental use was approved by the Animal Ethics Committee of Capital Medical University (personnel No. 15479). All of the surgeries were performed under anesthesia, and all efforts were made to minimize suffering. Antibiotics were administered to the animals to reduce the risk of post-surgical infection.

### Animal vocal fold wound model and the VFFs culture

The canines were anesthetized using an intramuscular administration of ketamine hydrochloride (50 mg/kg). The lamina propria was injured in 30 vocal folds of 17 canines by the localized resection of the anterior and middle portion of the vocal folds at a depth down to the vocal fold muscle under direct laryngoscopy using video monitoring. Four of the vocal folds were left uninjured for use as normal controls.

The isolation and culture of VFFs were performed according to the methods of Hirano [25] and Thibeault [26]. After the whole lamina propria of the anterior and middle portion of the vocal folds was resected, the removed vocal folds were cut into small pieces (1×1 mm^3^) and placed into cell culture dishes containing Dulbecco's modified Eagle's medium (DMEM; Gibco BRL, New York, USA) with 10% fetal bovine serum (FBS; Invitrogen, Carlsbad, NW, USA), 100 U/ml penicillin and 100 μg/ml streptomycin. The Petri dishes were maintained at 37°C in a 5% CO2 cell culture incubator. The medium was changed every 2 to 3 days until the cells had migrated from the explant. After the cells reached 80% to 90% confluence, the fibroblasts were subcultured.

### Culture of ADSCs and fibroblastic differentiation

The culture techniques were performed as described in our previous study [17]. Briefly, adipose tissue (approximately 8–10 g) was collected from the inguinal area, cut into small pieces, and then digested at 37°C for 40 minutes using 0.2% collagenase type I (Sigma-Aldrich, St. Louis, USA) and 2% bovine serum albumin (AbD Serotec, Kidlington, UK). Enzyme activity was neutralized with DMEM containing 10% FBS. The samples were filtered through a 100-μm nylon mesh to remove cellular debris and were incubated overnight at 37°C with 5% CO2 in DMEM containing 10% FBS, 100 U/ml penicillin and 100 μg/ml streptomycin. The medium was changed every 1 to 2 days. The subculture and identification techniques were described in our previous study [17].

The fibroblastic differentiation-inducing medium consisted of DMEM supplemented with 100 ng/ml connective tissue growth factor (CTGF; Biovendor, Brno, Czech Republic) and 50 μg/ml ascorbic acid (Sigma-Aldrich, St. Louis, USA) with 10% FBS, 100 U/ml penicillin and 100 μg/ml streptomycin. ADSCs cultured in normal DMEM were used as a control. The medium was changed twice each day. On days 7, 14 and 21, the dADSCs were collected for observation and cell immunofluorescence studies.

### Comparison of dADSCs and VFFs in vitro

The cell morphology and surface protein expression of the two types of cells were compared. Morphologic observations of the third passage dADSCs and VFFs were made using an inverted microscope. The cell surface protein expression levels were identified by cell immunofluorescence.

When the cells reached 80% to 90% confluence, they were fixed in ice-cold 4% paraformaldehyde for 10 minutes and rinsed with phosphate-buffered saline (PBS). They were permeabilized in PBS containing 0.3% Triton X-100 for 30 minutes at room temperature (RT). All cells were blocked (3% horse serum in PBS, 30 minutes, RT) and incubated for 2 hours (4°C) with the following primary antibodies: vimentin (monoclonal mouse vimentin, 1∶200; Novus, Littleton, CO, USA) and fibronectin (polyclonal rabbit fibronectin, 1∶100; Abcam, Cambridge, MA, USA). After a thorough washing, the cells were incubated with Alexa Fluor 488-conjugated goat anti-rabbit IgG (1∶400; Molecular Probes, Eugene, OR, USA) and 594-conjugated goat anti-mouse IgG (1∶400; Jackson ImmunoResearch Laboratories, West Grove, PA, USA) for 1 hour (RT). All slides were counterstained using Hoechst 33342 (1∶1000; Roche; 10 minutes, RT) for nuclear staining.

### Cell-scaffold complex construction and implantation

The cell-scaffold complexes were constructed as described in our previous study [17]. The three types of cells (dADSCs, ADSCs and VFFs) were trypsinized (0.05% trypsin; Sigma, USA), suspended in DMEM containing 10% FBS and subcultured at a concentration of 2,000 cells/cm2. Cultured cells were prepared by incubation with Try-EDTA for 5 minutes in a 37°C incubator. The cells were then washed with PBS. Using DAPI as a marker for the cells, we made a single cell suspension (3 ml) at a density of 1.0 × 105/cm2 and then inoculated the culture into hyaluronic acid gel (20 mg/ml, Changzhou Institute of Pharmaceutical Research, China) that had been soaked in DMEM for 5 minutes. After 20 minutes, DMEM was added parallel to the surface of the gel. Following co-culture in the gas-liquid interface for one week, the complexes were constructed and examined using a scanning electron microscope (SEM).

Seven days after the vocal folds were injured, we implanted the different cell-scaffold complexes (3–4 ml, 1.0×105 cells/ml) at the site of the local vocal folds locally injury under direct laryngoscopy. The experimental subjects were divided into four groups according to the implanted complex: the dADSCs implanted group, the ADSCs implanted group, the VFFs implanted group (N = 10 vocal folds in each group) and the normal control group (N = 4 vocal folds).

### Measurements of vocal fold morphology and lamina propria ECM proteins

At 15 days, 40 days, 3 months and 6 months after implantation, the morphologies of the vocal folds and glottal closure were examined using 0° endoscopy. Then, the canines were humanely sacrificed, and their larynges were carefully dissected. Consecutive coronal sections of 10 μm thickness were cut from each larynx on a freezing microtome (CM1850, Leica, Mannheim, Germany).

The expression levels of ECM proteins, including collagen, elastin, hyaluronic acid, decorin and fibronectin in the vocal fold lamina propria were evaluated on adjacent tissue sections. For each type of stain, the sections from all time points were stained simultaneously to ensure uniform conditions for subsequent quantitative analysis.

The sections were fixed in ice-cold 4% paraformaldehyde (10 minutes), rinsed in PBS, and permeabilized in PBS containing 0.3% Triton X-100 (30 minutes, RT). All reagents and incubations were performed in PBS containing 0.3% Triton X-100 and 0.3% horse serum. The sections were blocked (3% horse serum in PBS, 30 minutes, RT) and then incubated overnight (4°C) with the following primary antibodies: collagen (polyclonal rabbit collagen, 1∶200; Abcam, Cambridge, MA), hyaluronic acid (polyclonal sheep hyaluronic acid, 1∶50; Abcam, Cambridge, MA), elastin (polyclonal rabbit elastin, 1∶100; Abcam, Cambridge, MA), decorin (polyclonal rabbit decorin, 1∶100; Abcam, Cambridge, MA) and fibronectin (monoclonal mouse fibronectin, 1∶100; Abcam, Cambridge, MA). After thorough washing, the sections were incubated with Alexa Fluor 488-conjugated goat anti-rabbit IgG (1∶400; Molecular Probes, Eugene, OR), or goat anti-mouse IgG (1∶400; Molecular Probes, Eugene, OR) or donkey anti-sheep IgG (1∶400; Jackson ImmunoResearch Laboratories, West Grove, PA) for 2 hours (RT). Control sections in which primary antibodies or secondary antibodies were omitted showed no labeled cells. All sections were counterstained using Hoechst 33342 (Roche; 1∶1000; 10 minutes, RT) for nuclear staining.

**Figure 1:**
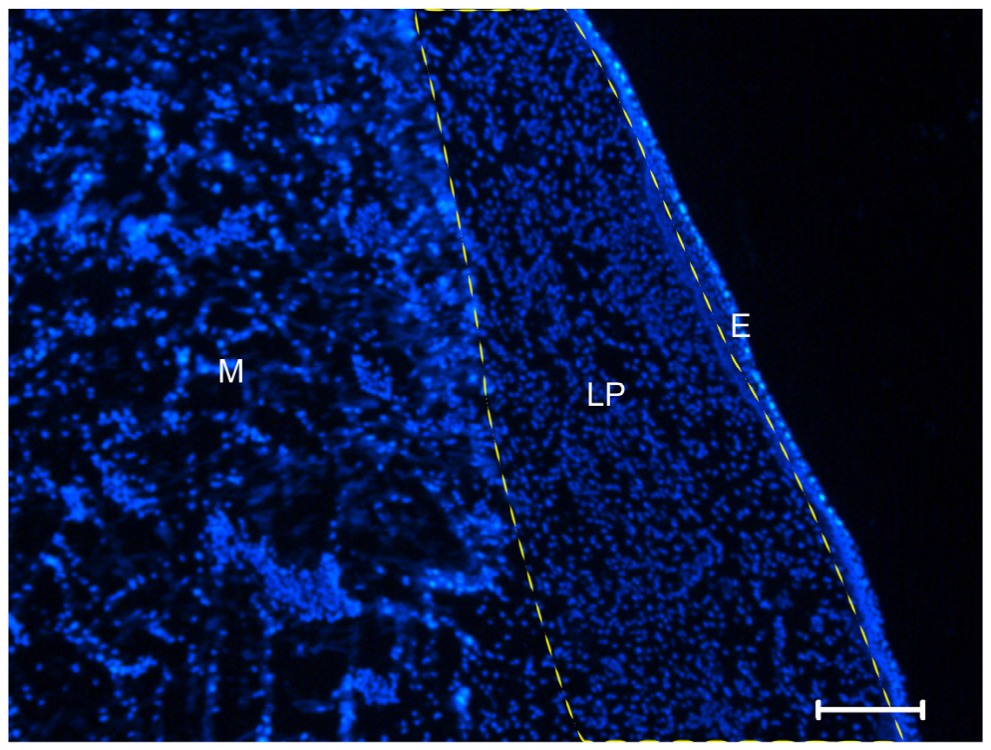
Diagram of the layered structure of the vocal fold (Hoechst staining). The canine vocal fold includes the epithelium (E), lamina propria (LP) and muscle (M). Scale bar  =  100μm.

To define the layered structure of the vocal folds in the study, we used the theory of Hirano [27] as a reference. For all of the animals, sections for analysis were collected at 100 μm intervals. Areas of interest with the vocal fold lamina propria (Figure 1), were observed with a Leica fluorescence microscope (DM 5000 B; Leica) with excitation and emission wavelengths of 470 and 525 nm (Alexa Fluor 488). Images were obtained with the Leica application suite (version 2.20) and analyzed with Leica imaging systems (Leica QWin Standard V2.2) and NIH ImageJ software [28]. The immunofluorescence intensity of selected areas of tissues (lamina propria) was evaluated with imaging software. All data were depicted as the mean±S.E.M. of three to five independent experiments for the different time points examined.

### Statistical analysis

Data were analyzed for statistical significance using ANOVA and appropriate post hoc tests (Student-Newman-Keuls or Kruskal-Wallis multiple comparison procedures, as appropriate) to determine significant differences between groups. A level of *p*<0.05 was considered to be statistically significant. The SPSS/PC 11.5 package was used for the statistical analysis of the data (SPSS Inc., Chicago, IL, USA).

## Results

### Comparison of dADSCs and VFFs in vitro

#### Cell morphology

**Figure 2:**
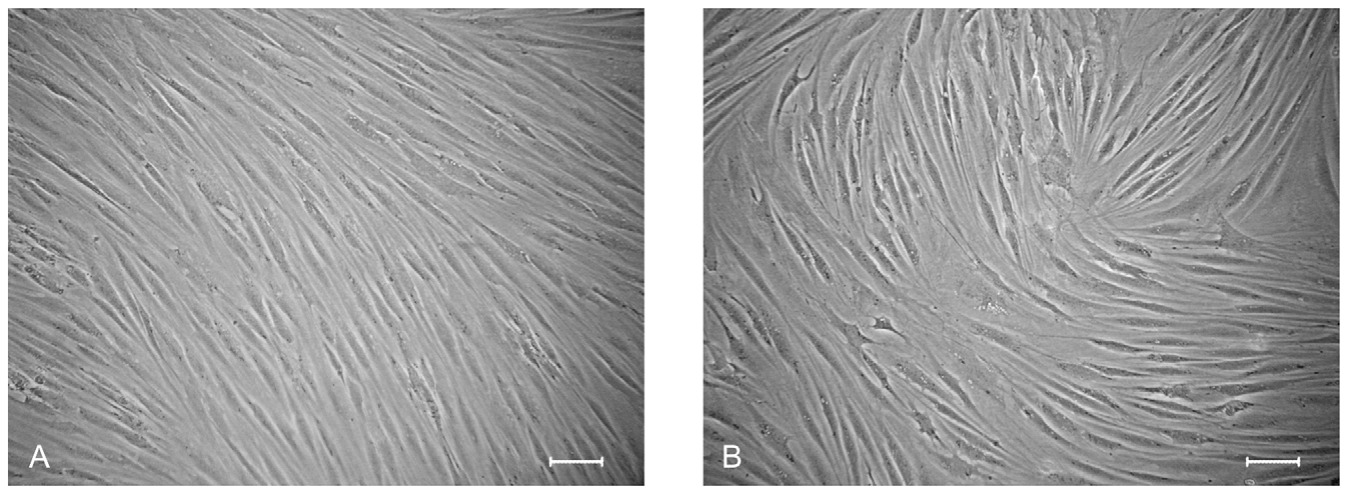
The cell morphology of vocal fold fibroblasts (VFFs). (A) and differentiated adipose-derived mesenchymal stem cells (dADSCs) (B). Both showed a spindle shape. Scale bar  =  100μm.

The VFFs showed a long spindle-like shape (Figure 2A). The ADSCs obtained from adipose tissues exhibited a whirlpool-like adherence with a vortex-like arrangement. Fourteen days after fibroblastic differentiation, the dADSCs were more homogeneously fibroblast-like and typically spindle shaped, with a fascicular arrangement (Figure 2B). The cell density was higher at day 21.

#### Cell immunofluorescence for the determination of surface-protein expression

**Figure 3:**
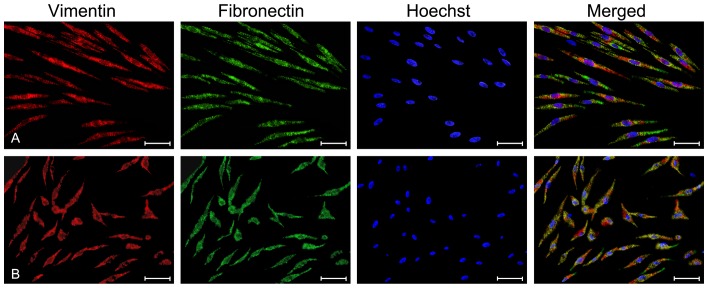
Cell immunofluorescence for the observation of surface protein expression. Both vocal fold fibroblasts (VFFs) (A) and differentiated adipose-derived mesenchymal stem cells (dADSCs) (B) can express vimentin and fibronectin. Scale bar  =  50μm.

Immunofluorescence cell staining demonstrated that the VFFs expressed vimentin and fibronectin, and the percentage of positive cells was more than 95.4% (Figure 3A). The dADSCs were also able to express vimentin and fibronectin, and the percentage of positive cells was more than 91.7% (Figure 3B). There was no significant difference in the percentage of positive cells between the VFFs and dADSCs (*p*>0.05).

#### Cell-scaffold complex features

Under SEM, one week after the three types of cells were mixed with the hyaluronic acid gel, they had permeated into the scaffolds and proliferated continuously. A large number of cells had attached to the scaffold material, migrating, proliferating and infiltrating the interior, where they continued to proliferate and differentiate.

### Changes in vocal folds after cell-scaffold complex implantation

#### Vocal fold morphological changes

Each group exhibited different changes according to the observation times. Fifteen days after implantation, all of the vocal folds were hyperemic and edematous, and a pseudomembrane was observed in parts of the vocal folds (dADSCs group 71.4%, ADSCs group 71.5%, and VFFs group 100%). Forty days after implantation, the hyperemia, edema and pseudomembrane had disappeared from the vocal folds. The site of implantation was slightly plump. Three months after implantation, the margins of the vocal folds were slightly irregular, but no obvious stiffness was observed. Six months after implantation, the surface of the vocal folds was almost smooth in the dADSCs group and the ADSCs group, reaching near-normal glottal closures. However, in the VFFs group, the vocal folds were slightly concave and stiff. There were no allergic or rejection reactions during the observation period.

#### Changes in vocal fold lamina propria ECM proteins

**Figure 4:**
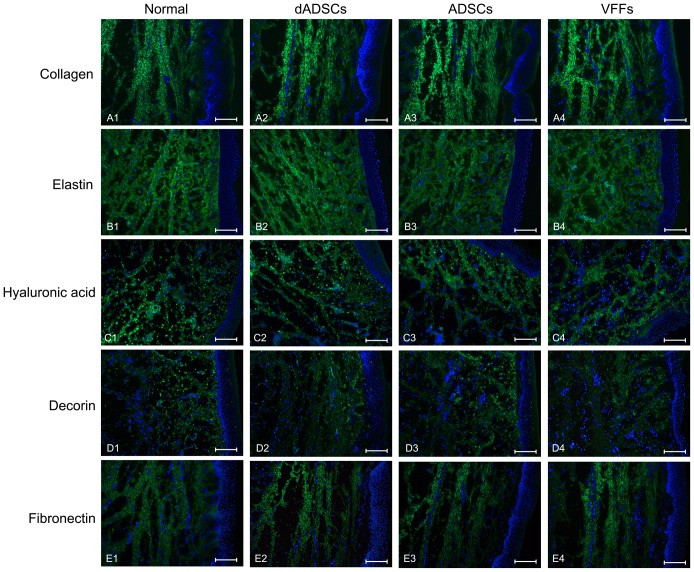
Photomicrographs showing the expression of extracellular matrix proteins. (A. collagen, B. elastin, C. hyaluronic acid, D. decorin, E. fibronectin) using immunofluorescent staining of the lamina propria of vocal folds in the normal control group (A1, B1, C1, D1, E1) and at 6 months after the implantation of differentiated adipose-derived mesenchymal stem cells (dADSCs) (A2, B2, C2, D2, E2), adipose-derived mesenchymal stem cells (ADSCs) (A3, B3, C3, D3, E3) or vocal fold fibroblasts (VFFs) (A4, B4, C4, D4, E4). Scale bar  =  50μm.

**Figure 5:**
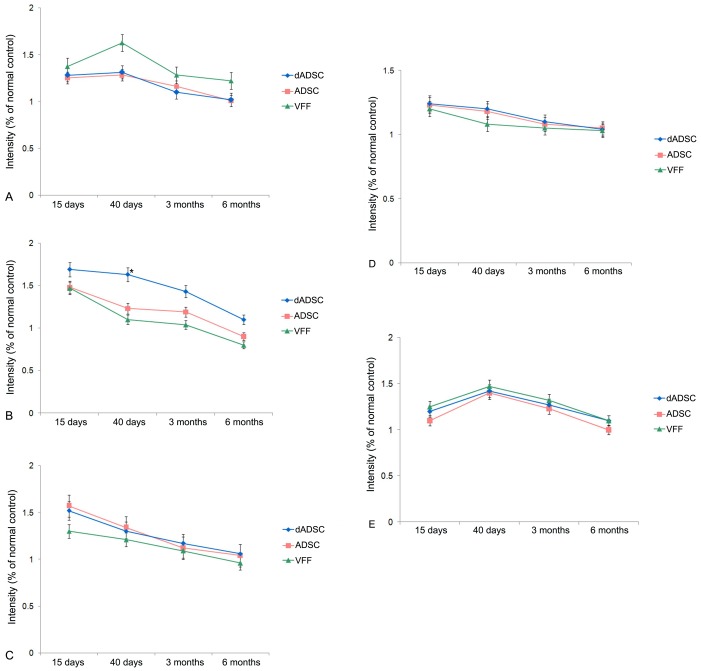
Histogram illustrating the gray value of extracellular matrix proteins. (A. collagen, B. elastin, C. hyaluronic acid, D. decorin, E. fibronectin) in the lamina propria of vocal folds from 15 days to 6 months after the implantation of differentiated adipose-derived mesenchymal stem cells (dADSCs), adipose-derived mesenchymal stem cells (ADSCs) or vocal fold fibroblasts (VFFs). Values are expressed as the mean±S.E.M. * indicates comparisons with the ADSCs and VFFs groups, significant difference (*p*<0.05).

*Collagen*. In the dADSCs and ADSCs groups, the changes in collagen were similar. The collagen was increased and scattered throughout all layers of the lamina propria from 15 to 40 days after the implantation, reaching a peak concentration at 40 days. Three months after the implantation, the collagen was decreased and concentrated in the middle and deep layers of the vocal fold lamina propria, although the arrangement was somewhat disordered. Six months after the operation, the fluorescence intensity of the collagen was similar to that of the normal controls; however, the distribution was slightly irregular. In the VFFs group, the increase in the fluorescence intensity of the collagen was more apparent during the 40 days after the procedure, at higher levels than the other two groups after 3 and 6 months, and with more disordered arrangement (Figure 4 A1-A4, Figure 5A).

*Elastin*. In the dADSCs group, elastin was distributed throughout all layers of the lamina propria for 15 days after the operation, and the local maximum values increased significantly (*p*<0.05, the dADSCs group versus the ADSCs group and the dADSCs group versus the VFFs group). Thereafter, the fluorescence intensity of elastin decreased and became concentrated in the middle layer of the lamina propria. Six months after the implantation, the fluorescence intensity and distribution of elastin were similar to those of normal controls. In the ADSCs and VFFs groups, the trend of variation in elastin was similar to that of the dADSCs group; however, the increment in elastin was lower than that in the dADSCs group (Figure 4 B1–B4, Figure 5B).

*Hyaluronic acid*. The trends in the variation of hyaluronic acid in the three implantation groups were similar. The fluorescence intensity of hyaluronic acid increased and was the highest within the 15 days after implantation; it was distributed throughout all layers of the lamina propria. From 40 days to 6 months later, the distribution of hyaluronic acid was limited to the superficial layer of the lamina propria, and the fluorescence intensity gradually decreased to the value observed in the normal controls (Figure 4 C1–C4, Figure 5C).

*Decorin*. The changes in decorin in the three implantation groups were similar. Within the first 15 days, the fluorescence intensity of elastin increased, but this was less apparent in the VFFs group. Decorin was distributed throughout all layers of the lamina propria. From 40 days to 6 months, the fluorescence intensity of decorin decreased and was limited to the superior layer of the lamina propria (Figure 4 D1–D4, Figure 5D).

*Fibronectin*. The fluorescence intensity and distribution of fibronectin over the three implanted groups exhibited a very similar variation. The fluorescence intensity of fibronectin increased and peaked at 40 days after the implantation and then decreased over the following 6 months exhibiting scattered expression throughout all layers of the lamina propria, which was similar to the expression pattern observed in the normal controls (Figure 4 E1–E4, Figure 5E).

## Discussion

In our study, we found that ADSCs could differentiate into fibroblast-like cells under the regulation of CTGF. The characteristics of the dADSCs were similar to those of the VFFs in vitro. We therefore used dADSCs in a vocal fold wound healing model in vivo, as they had the same morphology and cell protein expression levels. We observed a basically stable and gradual rational arrangement of the ECM proteins in the lamina propria and no scar formation in the morphology.

After Zuk et al. [29] discovered the MSC with the potential for differentiation into multiple cell types in human adipose tissue in 2001, ADSCs were widely used for tissue engineering. ADSCs can be successfully differentiated into adipocytes, osteoblasts, chondrocytes, neurocytes, and myocytes under the appropriate in vitro culture conditions. However, some features of mesenchymal stem cells are somewhat similar to those of fibroblasts, e.g., gene expression and cell markers, and they were not easy to distinguish [30], [31], making it difficult to identify the fibroblastic differentiation. CTGF is a member of the CCN protein family, which plays an important role in tissue repair, fibroblast proliferation and angiogenesis [32]. In 2010, Lee [23] used CTGF for fibroblastic differentiation from BMSCs for connective tissue healing. In 2011, Tong [24] used CTGF and polyester-based fibrous scaffolds to control the fibroblastic differentiation of human BMSC in vitro. In our experiment, we successfully induced ADSCs differentiation into fibroblast-like cells by regulating CTGF. We found that the morphology and arrangement of dADSCs in vitro were more similar to those of VFFs. The number of cells increased after 21 days, showing a satisfactory capacity for proliferation. Furthermore, we compared two types of cell surface proteins (vimentin and fibronectin) to determine whether the expressed proteins were similar between dADSCs and VFFs. Vimentin maintains the integrity and flexibility of cells and is expressed by mesenchymal cells. Fibronectin can be secreted by fibroblasts and plays a crucial role in wound healing. Our results also demonstrated that, similar to VFFs, dADSCs can express vimentin and fibronectin and further showed the dADSCs might have the characteristics of mesenchymal cells, indicating that they may play a major role in wound healing. However, the detailed capacities of dADSCs in vitro, such as their gene expression, particularly under conditions of vibration [33], were not examined in this study and thus remain to be examined in the future.

The construction of three-dimensional structural and functional cell-scaffold complexes has been a rapidly developing area of research in tissue engineering over the past 10 years. In this study, dADSCs, ADSCs and VFFs were used as seed cells. We used a hyaluronic acid gel i as a scaffold due to its ability to inhibit vocal fold scarring [34]. Based on the tissue engineering methods described in our previous study [17], we proposed three types of cells seeded in a hyaluronic acid gel using a gas-liquid interface co-culture method. After the implantation, we observed differences in the morphology and ECM protein expression between the three implantation groups.

In the early stage after implantation, less pseudomembrane was observed in the dADSCs and ADSCs groups than in the VFFs group, indicating that the dADSCs and ADSCs may possess greater anti-inflammatory capacity. In the late stages, the vocal folds of the dADSCs and ADSCs groups achieved an almost smooth surface, while the vocal folds were slightly concave and stiff in the VFFs group. This may due to the difference observed in the ECM proteins.

Collagen is the principal element in vocal fold scar formation, while the hyaluronic acid and decorin have the capability to inhibit collagen deposition and reduce scar formation [14], [15], [25], [34]. ADSCs may modulate the vocal fold scarring of fibroblasts by reducing collagen secretion and increasing hyaluronic acid secretion [35]. We believe that the structure of the complexes consisting of dADSCs or ADSCs within the hyaluronic acid scaffolds was more commonly inherent in the rationalization of the ECM distribution. After the implantation of the cell-scaffold complexes, the high levels of hyaluronic acid and decorin were able to create a favorable environment for vocal fold repair and regeneration which was suitable for lamina propria restoration; moreover, the proliferation of fibrous tissue was clearly inhibited in the early stages. Furthermore, we found that collagen was gradually reduced to near-normal levels and distributed in the deep layer of the lamina propria in the dADSCs and ADSCs groups in the middle and late stages. However, the collagen distribution was slightly irregular in the VFFs group. We considered that the VFFs' ability to secrete ECM proteins might decrease after culture in vitro and implantatation in vivo. The dADSCs and ADSCs showed their capabilities to simulate the orderly composition of ECM proteins, as described in previous studies [9], [18], [19]. This may be related to the role of regulatory factors and the microenvironment after injury, although further research is needed to understand the relevant effective mechanisms.

Elastin is known to play a significant role in promoting the recovery of vocal fold viscoelasticity. Thus, viscoelasticity can indirectly influence the vibration of the vocal folds. However, there were few changes in elastin, which was also examined in previous studies. In the current study, we found that the ability of dADSCs to secrete ECM proteins in the early period after implantation was stronger than that of the ADSCs and VFFs, particularly in terms of elastin secretion. The fluorescence intensity of elastin peaked at 15 days and was maintained at a high level for 3 months after dADSCs implantation. The use of the dADSCs may be more effective for the recovery of vocal fold vibration, which facilitates the functional recovery of the vocal folds. We believe that this function of dADSCs may be related to the characteristics they share with the resident VFFs. Moreover, the ability of mesenchymal stem cells to secrete ECM proteins which was inherited by the dADSCs, may contribute to the recovery of vocal fold vibration. However, further experiments are required to verify this function, e.g., quantitative detection of ECM protein secretion. Moreover, the measurement of viscoelastic properties and laryngeal high-speed photography are needed to evaluate vocal fold vibration in further studies.

Fibronectin effectively promoted wound healing during the early stages. As tissue repair continued, fibronectin promoted wound contraction, which is a critical step in wound healing. Thibeault [16] previously reported that fibronectin increases after cell-scaffold implantation. In our study, fibronectin secretion was similar in the three implantation groups. Fibronectin increased in the early period after the implantation and then decreased to a normal level, which was beneficial for maintaining the normal structure of the cells and healing the wound.

Although we used DAPI as a cell marker, we were unable to trace the cells after injection, potentially because the immunofluorescence intensity weakened with time, which was a limitation of our study. However, satisfactory cell markers are important for the detection of seed cell survival, distribution and differentiation and are also effective for analyzing ECM protein secretion. This area of focus will be improved and discussed in further studies.

## Conclusions

In this study, ADSCs were induced to differentiate into fibroblast-like cells. The dADSCs have the ability to regulate the generation and orderly distribution of ECM proteins for promoting vocal fold wound healing. Moreover, the dADSCs had a stronger ability to secrete elastin when compared with ADSCs and VFFs, which may be beneficial for vocal fold functional vibration recovery. Although the repaired vocal folds still differed from the normal controls, this technology provides an optimistic prospect for the treatment of vocal fold wound healing.
